# Strain-induced magnetic domain wall control by voltage in hybrid piezoelectric BaTiO_3_ ferrimagnetic TbFe structures

**DOI:** 10.1038/srep23038

**Published:** 2016-03-18

**Authors:** Olivier Rousseau, Raphael Weil, Stanislas Rohart, Alexandra Mougin

**Affiliations:** 1 Laboratoire de Physique des Solides, CNRS, Univ Paris-Sud, Université Paris-Saclay, 91405 Orsay Cedex, France

## Abstract

This paper reports on the voltage dependence of the magnetization reversal of a thin amorphous ferromagnetic TbFe film grown on a ferroelectric and piezoelectric BaTiO_3_ single crystal. Magneto-optical measurements, at macroscopic scale or in a microscope, demonstrate how the ferroelectric BaTiO_3_ polarisation history influences the properties of the perpendicularly magnetized TbFe film. Unpolarised and twinned regions are obtained when the sample is zero voltage cooled whereas flat and saturated regions are obtained when the sample is voltage cooled through the ferroelectric ordering temperature of the BaTiO_3_ crystal, as supported by atomic force microscopy experiments. The two steps involved in the TbFe magnetization reversal, namely nucleation and propagation of magnetic domain walls, depend on the polarisation history. Nucleation is associated to coupling through strains with the piezoelectric BaTiO3 crystal and propagation to pinning with the ferroelastic surface patterns visible in the BaTiO3 topography.

In order to reduce energy consumption of magnetic information technologies, there is a considerable interest in the control of magnetic properties through voltage instead of electrical current. Indeed, controlling the magnetization of a ferromagnetic, an antiferromagnetic or a ferrimagnetic layer with electric fields would allow the conception of electrically writable and magnetically readable memories. Electric fields effects on magnetism can be driven by either purely electronic or electrostatic effects or occur through strain coupling to a ferroelectric material^1^. Various systems are investigated for the electrical control of magnetic properties like anisotropy, spin order, ordering temperature or domain structure. In magnetic metallic systems, electric fields are rapidly screened. Nevertheless charges induced at a metal/dielectric interface can drive electronic and electrostatic magnetic modifications, such as changing the surface magnetic anisotropy^2^,^3^, or inducing resonances^4^. A bistable magnetization switching by precessional magnetization dynamics using electric field pulses has even been observed in ultra-thin nanoscale ferromagnetic cells^5^. Multiferroic materials, wich are almost insulators, could be ideal systems,, since they exhibit an intrinsic magnetoelectric coupling between magnetic and electric orders^6^. However, as most multiferroelectric materials are antiferromagnets, addressing a net magnetization with an electric field was done combining the magnetoelectric coupling between antiferromagnetism and ferroelectricity with exchange coupling between a ferromagnet and the antiferromagnetic order^7^,^8^,^9^,^10^,^11^.

Additionally to that, many results were obtained in hybrid artificial multiferroic structures based on thin magnetic layers deposited on top of a layer which is both ferroelectric and piezoelectric. In rare systems, a direct coupling between magnetic orders and pure charge effects was evidenced; an illustrative case is that of a carrier-mediated ferromagnet La_0.8_Sr_0.2_MnO_3_ grown on Pb(Zr_0.2_Ti_0.8_)O_3_ that exhibits a shift of the ordering temperature due to change in valency induced by charge carrier modulation^12^,^13^. In hybrid piezoelectric and ferroelectric systems, the key idea is to provoke a strain transfer from ferroelastic domains existing in the ferroelectric media into continuous magnetostrictive films grown on top. This is quite successful but in many systems, it is quite difficult to disentangle the combined effects of electrostatics from those of ferroelasticstrains^14^,^15^,^16^,^17^,^18^,^19^. Our choice for the artificial multiferroic structure is a ferrimagnetic alloy of Tb and Fe coupled with a ferroelectric crystal of BaTiO_3_ (BTO). Ferrimagnetic heavy Rare Earth (RE)- Transition Metal (TM) alloys drew a great deal of investigation in the 1970’s due to their application in magneto-optical data storage^20^. Ferrimagnetism refers here to the anti-parallel coupling of the TM sub-lattice magnetization with that of RE sub-lattice. Both magnetizations may compensate each other and a tuning of the alloy composition allows working with a very low magnetization^21^. There was a recent renew of interest for such ferrimagnets that exhibit peculiar dynamics^20^,^22^, anisotropy^23^,^24^ and spin torque efficiencies^25^ in the vicinity of compensation. Bulk BTO crystals were also extensively analysed^26^ and largely used as ferroelectric substrates to grow hybrid devices because of the BTO order temperature which is just above room temperature. Several ferroelastic and ferroelectric domain arrangements may be observed depending on the thermal and electrical history of the BTO^19^. The balance of *a*-domains (in-plane polarized) and *c-*domains (out-of-plane polarized) can be altered by applying a voltage *V* or by changing the temperature, leading to characteristic top surface patterns induced by the underlying ferroelastic patterns.

In this report, we show how the history of perpendicular polarisation of a ferroelectric BTO crystal influences the magnetic properties of the top deposited TbFe film. With the proper annealing procedure, when cooled with zero applied electric field, the magnetization reversal of TbFe requires a higher magnetic field than when cooling is performed with an electric field that saturates the polarisation state of the BTO. A remnant saturated and flat BTO interface always promotes easier nucleation and propagation of magnetic domains. These effects result from the magnetoelastic couplings of TbFe magnetic domains to the underlying BTO ferroelastic structure.

## Results

### BTO and ferroelectricity

**Figure 1:**
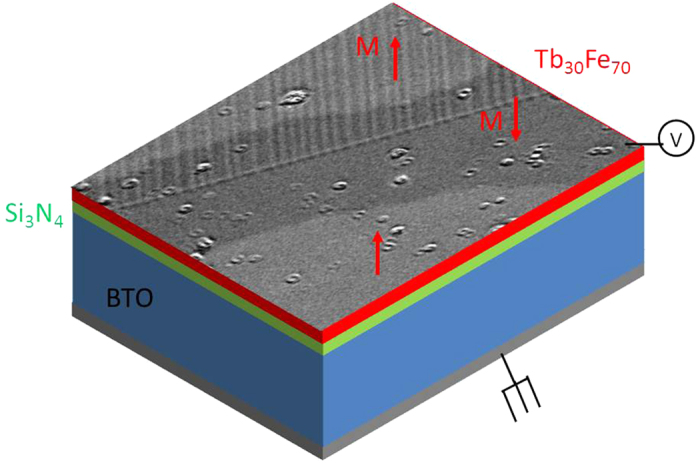
Sketch of the stack of the device. The top image is a magnetic domain structure, measured by Kerr microscopy in the TbFe top film. We studied the influence of the BTO state controlled by a voltage V applied perpendicularly to the sample plane between the TbFe top electrode and the bottom of the BTO single crystal.

**Figure 2:**
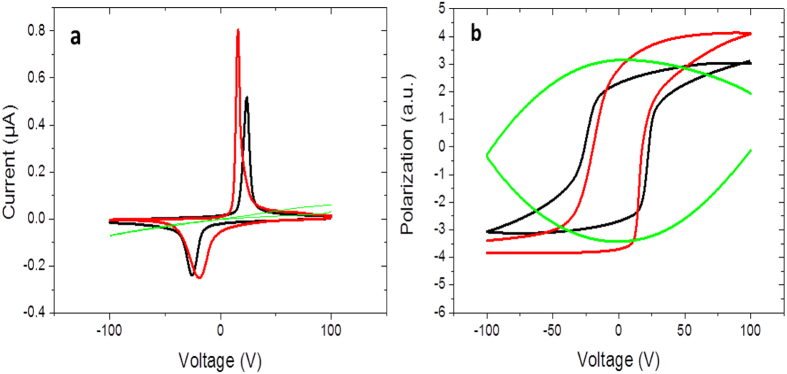
Polarisation reversal of BTO. (**a**) Typical current flowing through the BTO during voltage loop. (**b**) Electric polarisation obtained by integration of the current loop. The black curve is at room temperature, the red one at 90 °C and the green one at 140° where BTO is no more ferroelectric.

Figure 1 represents a sketch of the device stack, which allows the application of voltage across the BTO substrate to tune its ferroelectric state (see experimental section for more details). Figure 2(a) shows the current flowing through the BTO while the voltage is swept. Peaks correspond to the reversal of the perpendicular polarisation of the BTO and define the coercive voltage. This coercive voltage decreases when temperature is increased: it is about 25 V at room temperature, drops down to about 20 V at 90 °C and vanishes at the ferroelectric Curie temperature T_FE_. At 140 °C, there is no more polarisation reversal as shown in Fig. 2(b). T_FE_ has been measured to be 134 °C, in good agreement with literature^26^.

**Figure 3:**
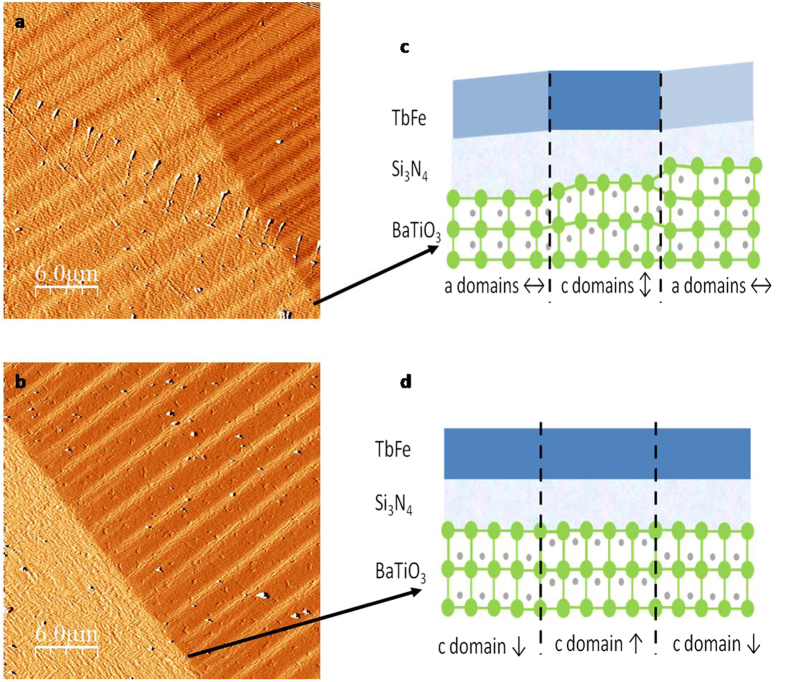
AFM image and the sketch of the corresponding structure. AFM images of the BTO/TbFe sample. In (**a**) the voltage while cooling from 140 °C was 0 V, in (**b**) it was 100 V. The contrast of the image is the derivative of the topography^27^. Each colour corresponds to a different slope of the substrate. The two scanned areas are slightly shifted between the two images. The images have been recorded at the separation area between low and high domain wall velocity regions of Fig. 5. Sketch of possible relevant different BTO states depending of the polarisation history: for 0 V cooling (c) mixture of *a* (in-plane) and *c* (out-of-plane) domains in the BTO substrate thats organizes in micron-scaled stripes; for 100 V (d) “pure” *c* domains regions exhibiting a flat surface. Different strains states are expected in top grown layers, indicated by different colours in the TbFe.

BTO shows ferroelectric as well as piezoelectric properties. Next we investigate the BTO structure by atomic force microscope (AFM) (Fig. 3(a,b)), driving temperature back and forth through the ferroelectric Curie temperature T_FE_, as a function of the applied voltage. Indeed, when BTO is cooled from 140 °C to a temperature where it is ferroelectric, two different states can be achieved, depending on the voltage applied during the cooling through T_FE_. If a 0 V voltage is applied, a multi-domain in-plane polarisation state (Fig. 3(c)) can be expected as 0 V will oppose to the formation of out-of-plane polarized domains (*c* domains). On the contrary if a perpendicular voltage larger than the coercive voltage is applied, e.g. 100 V (any non-zero voltage should work as near T_FE_ the coercive voltage is vanishing), a uniform perpendicularly polarized BTO is expected (Fig. 3(d)). Two characteristic AFM derivative images^27^ and sketch of their expected corresponding different BTO states are represented in Fig. 3. The alternation of brighter and darker areas at the scale of 1 μm in Fig. 3(a) (0 V cooling) demonstrates indeed that the BTO is in the twinned state everywhere as expected from a cooling with 0 V (Fig. 3(c)). A saw-tooth roof pattern is observed in the unpolarised regions, referred as twinned state in the following. On the contrary, after a polarisation voltage is applied during cooling, the BTO is uniformly perpendicularly saturated and hence flat as shown in Fig. 3(b,d). Moreover, at small scales, it is known^19^,^26^ that a mixture of *a* and *c* polarisation and strain domains in the BTO substrate organize in micrometric stripes whereas “pure” *c* domains regions exhibit a flat surface. We don’t have any direct observation of the polarisation domains in the BTO but the induced surface supports strongly this conclusion, in agreement with all previous reported work on BTO. It is important to notice that the twinned state, once erased by a saturating voltage, cannot be recovered by decreasing the voltage down to 0 V or any depolarisation procedure based on an out-of-plane applied electrical field at a constant temperature. Such procedure forms a flat state with up and down *c* domains as a pattern for the zero net polarisation. A thermal cooling under 0 V is necessary for the BTO to be twinned everywhere. At larger scale, there are also minor areas, as the top right area in Figs 2 and 3, which remain in a twinned state whatever the temperature and voltage schemes because they are related to defects. Those areas are visible by naked eye, they are already present when the substrates are delivered and can be generated, and modified by any mechanical strain, like the one induced by tweezers during sample manipulation.

### Magnetization characterization

**Figure 4:**
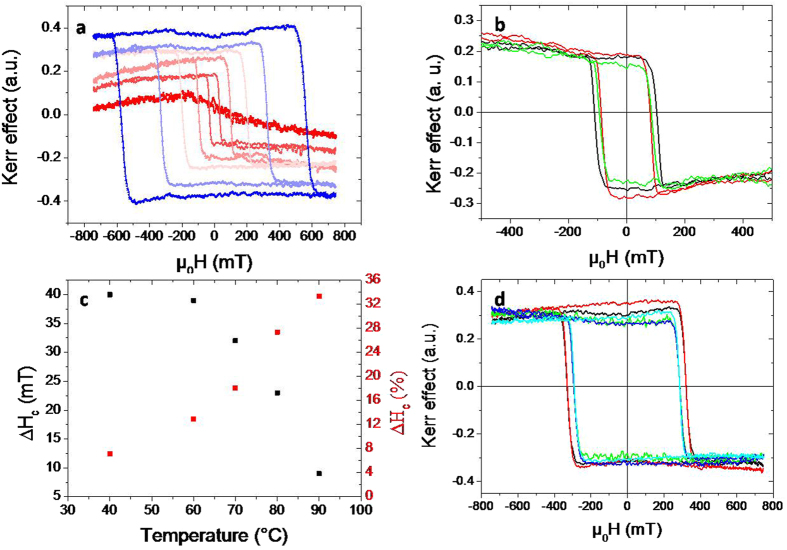
Hysteresis loops, coercive field and BTO states. (**a**) Polar Kerr hysteresis loops as function of the applied magnetic field at 40 (blue), 60, 70, 80, 90 °C and 100 °C (red). The coercive field decreases with increasing temperature. (**b**) Hysteresis loops at 80 °C for different voltage histories. The sample is heated up to 140 °C and then cooled down to 80 °C while a continuous voltage is applied of 0 V (black) −100 V (red) or 100 V (green). (**c**) Difference of coercive fields between the twinned and flat BTO as a function of measurement temperature in absolute value (black) and relative (red). (**d**) Verification of the effect robustness at 60 °C: first the sample is cooled down to 60 °C, while applying 0 V (black); the sample is cooled to 20 °C and heated back to 60 °C (red); then, successively 100 V, 0 V and −100 V were applied for the green, blue, cyan curves.

We use magneto-optics, namely Kerr effect^28^, to investigate the TbFe film properties as a function of an external magnetic field perpendicular to the sample’s plane and for different temperatures. Hysteresis loops and domain wall structures were obtained by magnetometry with a red laser and by using a microscope^29^. Figure 4(a) shows the polar Kerr signal of TbFe on BTO as a function of the applied magnetic field and for different temperatures. This signal, averaged over a spot about 1 mm^2^ in surface, gives the out-of-plane component of the Fe magnetization^30^. At room temperature, the magnetic anisotropy field of the film, proportional to the ratio between the anisotropy constant over the total magnetization, is so large that it was not possible to cover the entire film hysteresis loop experimentally. At higher temperatures, anisotropy is smaller and square loops can be measured as can be seen in Fig. 4(a). They exhibit a full remnant magnetization at zero field which supports a perpendicular anisotropy for the 7 nm thick Tb_30_Fe_70_ alloy film, over the entire temperature range up to the Curie temperature, (T_C_) determined to be about 100 °C.

Next, we focus on the TbFe hysteresis loops and magnetization reversal for the two different BTO states described in Fig. 3. First, we heat BTO/TbFe stack up to 140 °C (above T_FE_ and T_C_) to erase both magnetic and electric histories. BTO states are tuned under controlled electrical field cooling. TbFe hysteresis loops were recorded as a function of magnetic field after cooling the sample from 140 °C to the measurement temperatures under a constant voltage of 0 V, 100 V or −100 V. Figure 4(b) shows the measured TbFe hysteresis loops. Firstly, the major feature is that the loop corresponding to the 0 V cooling, which presents a mixed state of *a* and *c* domains, is clearly wider than the others, which present *c* domains only. No significant amplitude difference is measured, meaning that there is no significant change of the saturation magnetization. Secondly, both 100 V and −100 V cooling procedures give the same coercive field. This rules out any electric field (odd with the polarisation reversal) as well as any subsequent magnetoelectric coupling between the BTO and the ferrimagnetic film. This was expected from the presence of the Si_3_N_4_ underlayer and the 7 nm thickness of the TbFe film. The difference in coercive fields between the two BTO states, twinned surface and an out-of-plane saturated and thus flat surface, is represented in Fig. 4(c) and is in agreement with a change of perpendicular anisotropy due to strain difference between the two BTO states. The difference of coercive field increases with decreasing temperature. Of course the total perpendicular anisotropy becomes larger too when temperature is decreased reducing the relative strength of the strain mediated anisotropy. Lastly it is interesting to wonder about the robustness of the anisotropy change with thermal (below T_C_ and T_FE_) and voltage loops. This is what is represented in Fig. 4(d). A twinned BTO state was prepared by a 0 V cooling from above T_FE_ and the superimposed black and red curves show that cooling and heating back, with 0 V applied does not affect the coercive field as it does not affect the BTO state. However, keeping temperature constant, as soon the BTO is fully perpendicularly polarized keeping temperature constant by a 100 V saturating voltage (green curve), the coercive field decreases down to the value corresponding to a uniform and flat BTO state. When the BTO polarisation is further reversed at this constant temperature, it goes through a state with a zero net polarisation pattern with up and down c domains, with the small remnant Hc. This is why the coercive field of TbFe is not affected by a perpendicular voltage loop at constant temperature, as shown in blue and cyan curves of Fig. 4(d). All the features described above happen over the entire temperature range where hysteresis loops can be easily obtained, from 40 °C to 90 °C. It is important to note that the low coercive field (H_C_) constitutes a remnant state that does not depend on the electrical field value anymore. As said before, the only way to recover a higher H_C_ is to heat the sample above T_FE_ and then cool it under 0 V towards the twinned BTO state shown in Fig. 3. The coupling between the ferrimagnetic metallic film and the BTO crystal is the consequence of the imprint of BTO ferroelastic/ferroelectric *a* and *c* domains in the TbFe magnetic ones. It is natural to wonder how the TbFe magnetization reversal occurs on top of the different BTO states and in particular to see if magnetic domain wall nucleation and subsequent propagation are differently affected. We demonstrate below that the ferroelastic BTO states are indeed essential in both magnetic domain nucleation (related to the anisotropy field) and propagation (hindered by pinning) in the perpendicular TbFe film.

### Imaging of magnetic domain reversal

**Figure 5:**
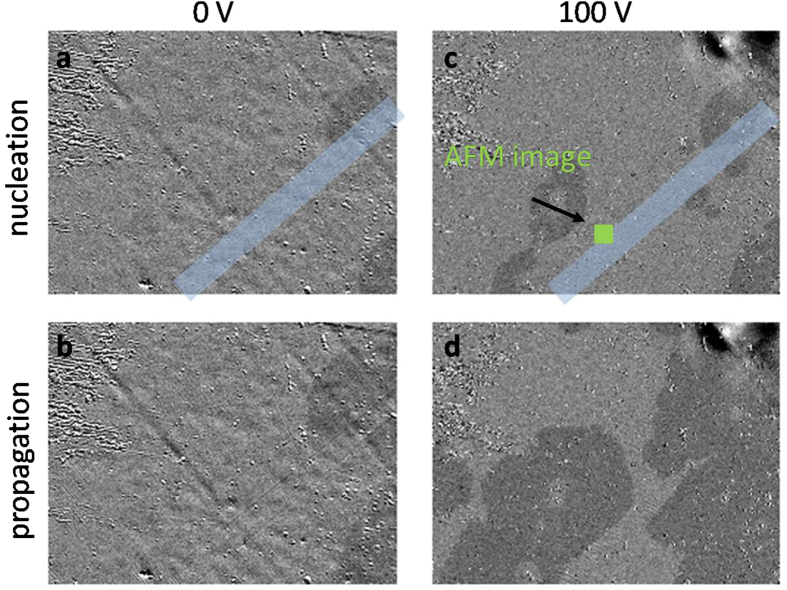
Polar magneto-optical images of nucleation and propagation. The images taken at 80 °C showing the magnetic domains developing in the TbFe film for the two BTO states: 0 V cooling (twinned state - left column) images (**a**,**b**) and 100 V cooling (saturated - right column) images (**c**,**d**). Starting from the magnetic saturation of the TbFe film, one single domain nucleation procedure was applied for the distinct BTO states: 4 magnetic pulses of 100 mT during 250 ms. (**a**,**c**) images show magnetic domains just after nucleation. A common propagation procedure was then applied, 20 additional magnetic pulses. The obtained magnetic domains are in (**b**,**d**) respectively. The images are 895 μm by 680 μm. The light blue areas correspond to the permanent twinned state area.

To determine the magnetization reversal mechanism, as a function of the BTO state, we performed Kerr microscopy measurements. In magnetic systems with an out-of-plane magnetization and exhibiting remnant square hysteresis loop, the nucleation field fixes the beginning of the reversal. A full reversal occurs more or less rapidly by the subsequent domain wall propagation and the time required for a complete reversal depends on the applied propagation field. Note that in square loops like ours, the time required for magnetization reversal does not change the coercive field which is given by the initial magnetization reversal at the nucleation field. Figure 5 shows a selection of magneto-optical images during TbFe magnetization reversal for the two BTO states, at 80 °C: 0 V cooling (twinned state – left column) and 100 V cooling (saturated - right column). First, nucleation is investigated. The same nucleation procedure, magnetic pulses, is used for the two states. By comparing images (a) and (c), it is obvious that nucleation areas are created more efficiently in (c) corresponding to the case where the BTO had been saturated by an electrical field. There is no change in the position of the nucleation centres but depending on the BTO state, those positions exhibit different critical fields for their activation. This supports the hypothesis of global modification of the magnetic anisotropy amplitude by the BTO strain states, since the magnetization is not modified as seen in hysteresis measurements. Secondly, propagation was analysed. Growth of the nucleated magnetic domains was induced using magnetic pulses of 100 mT during 250 ms. As expected with the previously discussed hysteresis loops, which are square in both BTO states and averaged on millimetre sizes, TbFe reversal occurs by domain nucleation followed by a fast domain wall propagation. Both nucleation and propagation fields are smaller in the flat regions: the domain wall velocity is about 25 μm · s^−1^, roughly 3 times faster than in twinned regions.

**Figure 6:**
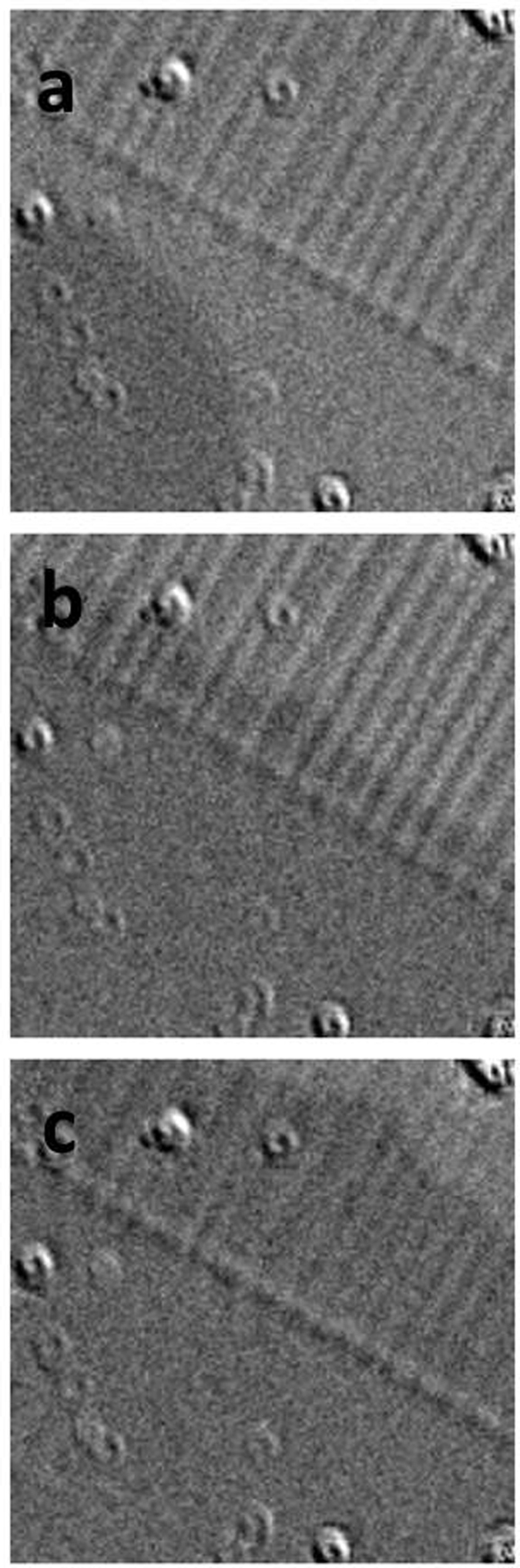
Polar magneto-optical images at 80 °C. The imagesshow the magnetic domains developing in the TbFe film with two BTO states in the field of view. (**a**) Nucleation of the reversal. (**b**) Propagation after 4 pulses of 120 mT and with 250 ms of duration (**c**) after 7 additional pulses. The images are 100 μm by 100 μm.

Figure 6 presents images at larger magnification so that twinned and flat saturated areas are clearly visible. Again nucleation happens in the uniform area, not in the border between the two BTO states. After the flat area is already fully reversed, the magnetization of the twinned part is further reversed by domain wall propagation, much slower, if additional field pulses of the same strength are applied. This difference of velocity changes the domain wall roughness: from a unique nucleation centre, in the flat area, a regular magnetic bubble forms with a smooth domain wall. When that domain wall enters the twinned region, it slows down and its interface becomes much rougher. This demonstrates that the domain wall is sensitive to the underlying pinning. This behaviour is in agreement with observations performed in other perpendicular materials in the thermally activated creep regime^31^.

## Discussion

We have demonstrated that the voltage induced changes of BTO state impacts on the magnetization reversal dynamics in full agreement with the macroscopic square hysteresis loops. The voltage applied during cooling from a non-ferroelectric state promotes the existence of *a* (0 V) or/and *c* domains (100 V). Our observations support a weaker perpendicular anisotropy of TbFe on *c* domains than on mixed *a* and *c* domains regions. Once BTO is polarised out-of-plane with only *c* domains and no in-plane a domains, the strain state cannot be modified by a perpendicular applied electrical field (constant or series of pulses) at fixed temperature. We believe that an in-plane electric field would produce *a* in-plane domains and allow the control of magnetization reversal properties through pure voltage control at fixed temperature. Magnetization reversal occurs by nucleation and propagation of domain walls, whatever the area. The coercive field is mainly given by the nucleation field. The uniform and flat parts are associated with a small nucleation field. In twinned parts on rough BTO surfaces, nucleation is harder. In many systems, roughness and structural defects have an opposite impact on nucleation: roughness induces additional nucleation sites and therefore a reduction of the nucleation field. Here, the important feature to take into account is not the BTO roughness but the fact that the latter is generated by a mixture of ferroelastic domains (Fig. 3(c)) that further induces different anisotropy in TbFe. As usual, roughness (the periodic twinned state) or defects slow down propagation and for the same magnetic field.

Temperature dependent magnetoelastic coupling has been observed in in-plane magnetic films directly deposited on BTO. In SmCo films, the thermal BTO structural phase transitions modify magnetic coercivity but seem to have no impact on the magnetic domain patterns^23^. In BTO/Fe, electric control of coercitivity was associated to anisotropy and magnetization changes^14^ or different symmetry of anisotropy evidenced macroscopically in association with the interface lattice distortion^32^. It is interesting to note that, unlike studies in other materials^15^,^19^, our measurements concern an amorphous ferromagnetic material that is solely influenced by the ferroelastic domains of the BTO. However, quantification of the change in their strain might be difficult^33^. Here, the coupling persists through the Si_3_N_4_ buffer layer. It is favoured by significant magnetostriction in such amorphous alloys, as measured in thicker films^34^.

In conclusion, we investigated the influence of the ferroelastic state of a BTO substrate on the magnetization reversal of an amorphous ferromagnetic TbFe alloy. By heating the samples above T_FE_ and T_C_, ferroelectric and ferromagnetic properties can be rewritten as new during a cooling with the appropriated voltage applied across the BTO. When 0 V is applied, a twinned surface resulting from mixed *a* (in-plane) and *c* (out-of-plane) polarisation domains, is favoured. On this twinned surface, perpendicular anisotropy, nucleation and coercive fields are increased, whereas the propagation velocity of the walls is decreased. When the sample is out-of plane polarized by a saturating voltage, it consists only of *c* domains with a flat surface: both nucleation and propagation field are smaller in this uniform strain surface and magnetization reversal becomes easier. A very promising experiment to achieve a full electrical control of TbFe properties at a given temperature could rely on a device in which an in-plane electric field could also be applied. This would allow driving the BTO polarisation in-plane, with no need of thermal cycling. Our results nevertheless demonstrate the ability to influence magnetization properties of amorphous layers with an electric field through a ferroelectric and ferroelastic substrate.

## Methods

The investigated TbFe film is a 7 nm thick Tb_30_Fe_70_ alloy (TbFe) deposited by e-beam co-evaporation of Tb and Fe in ultra-high vacuum (base pressure of 10^−10^ mbar) on a 5*5*0.5 mm^3^ commercial BTO (001) single crystal. Before the deposition of the ferrimagnetic alloy, a 23 nm thick Si_3_N_4_ was deposited by sputtering to prevent oxidation of the TbFe by the oxygen of the BTO. Without this insulator layer free of oxygen, any thermal anneal would continuously and irreversibly modify the magnetic properties of the ferrimagnet. The Si_3_N_4_ also promotes amorphous TbFe growth on BTO crystals. To prevent oxidation from the oxygen of air, a 5 nm thick capping layer of Al is deposited by e-beam evaporation just after the ferrimagnet growth. The BTO crystal is pasted with silver paste to a heating sample holder.

## Additional Information

**How to cite this article**: Rousseau, O. *et al*. Strain-induced magnetic domain wall control by voltage in hybrid piezoelectric BaTiO_3_ ferrimagnetic TbFe structures. *Sci. Rep.* **6**, 23038; doi: 10.1038/srep23038 (2016).
